# The Effects of a Mixture of Monochromatic Green and Blue Light on Growth Performance and Immune Response in Bursa of Fabricius by Morphometry Using Staining and Immunohistochemistry in Broiler Chickens

**DOI:** 10.3390/ani16081238

**Published:** 2026-04-17

**Authors:** Loredana Horodincu, Victor Cotrutz, Radu Herțanu, Adriana Petrovici, Ivona Popovici, Gheorghe Solcan, Alexandra Ciubotariu, Mădălina Henea, Lenuța Galan, Rareș Pogoreanu, Adina-Ștefana Dinuț-Cebuc, Silviu Stafie, Carmen Solcan

**Affiliations:** 1 Department of Preclinical Sciences, Faculty of Veterinary Medicine, “Ion Ionescu de la Brad” Iasi University of Life Sciences, 700490 Iasi, Romania; loredana.horodincu@iuls.ro (L.H.); adriana.petrovici@iuls.ro (A.P.); ivona.popovici@iuls.ro (I.P.); alexandra.ciubotariu@iuls.ro (A.C.); madalina.henea@yahoo.com (M.H.); lenuta.galan@iuls.ro (L.G.); carmen.solcan@iuls.ro (C.S.); 2 Greentek Lighting SRL, 700265 Iasi, Romania; victor.cotrutz@greentek.eu (V.C.); radu.hertanu@yahoo.com (R.H.); rares.pogoreanu@student.tuiasi.ro (R.P.); adina.dinut@greentek.eu (A.-Ș.D.-C.); silviu.stafie@greentek.eu (S.S.); 3 Faculty of Electronics, Telecommunications and Information Technology, “Gheorghe Asachi” Technical University of Iasi, 700506 Iasi, Romania

**Keywords:** broiler chickens, innovative housing, immune response, growth performance, melatonin receptors

## Abstract

The use of colored LED lights is a tool for controlling the development of lymphoid organs and the immune system in general. This study aims to analyze the effects of using simple and combined colored LED lights throughout a 6 week period (1–42 days of age). In this study, 336 one-day-old chicks were used, separated randomly into four groups with different sex and lighting systems, with each group being divided into four separate replicates (4 × 21 birds). The chicks in the WL-Male and WL-Female were exposed to white LED light (WL, 400–760 nm) for 6 weeks, while the chicks in the G-GxB-BL-Male and G-GxB-BL-Female were exposed to a combination of monochromatic lights as follows: green (560 nm) from 1 to 14 days of age, green and blue (480–560 nm) for 15–28 days of age, and blue lights (480 nm) for 29–42 days of age. The use of a mixture of green and blue LED lights (G-GxB-BL) resulted in a significant decrease in the average daily feed intake and feed conversion ratio compared to white light, without causing changes in the body weight of the chicks, average daily gain, mortality rate, and coefficient of variability. G-GxB-BL lights also improved the morphological development of the bursa of Fabricius (BF) compared to white light by significantly increasing the organ index and the lymphoid follicle area. At the same time, G-GxB-BL light compared to white light improved B lymphocytes proliferation in the BF by significantly increasing the lymphocyte density in lymphoid follicles, as well as the number of PCNA-positive cells. This light treatment had these results due to the activation of melatonin receptors, which led to a significant increase in Mel1a-positive cells and a significant decrease in the number of RORα-positive cells. These results demonstrate that G-GxB-BL lights improved the growth performance and immune response in the BF of broiler chickens.

## Simple Summary

It is known that green and blue LED lights have the power to modulate the immune response and growth performance in broiler chickens. However, the impact of these lights on the morphostructure of bursa of Fabricius (BF) has not been reported until now. In this study, 336 one-day-old broiler chickens were separated randomly in four groups with different sex and lighting systems, with each group being divided into four replicates (21 chickens per replicate). The chicks in the WL-Male and WL-Female groups were exposed to white LED light (WL, 400–760 nm) for 6 weeks, while the chicks in the G-GxB-BL-Male and G-GxB-BL-Female were exposed to a combination of monochromatic LED lights as follows: green (560 nm) from 1 to 14 days of age, green and blue (480–560 nm) for 15–28 days of age, and blue lights (480 nm) for 29–42 days of age. Indices for growth performance, organ index for the BF; immune organ morphology; and immunolabeling of proliferating nuclear antigen (PCNA), membrane receptor for melatonin (Mel1a) and nuclear receptor for melatonin (RORα) were determined. The results show that, compared to white light, our proposed light treatmeant improves growth performance and enhances the immune response in the BF.

## 1. Introduction

The bursa of Fabricius is a central and unique pillar of the avian immune system, being the primary lymphoid organ responsible for generating B lymphocyte diversity and establishing humoral immune competence [1]. Unlike other species, in birds, the BF provides the indispensable microenvironment in which non-thymic hematopoietic stem cells, colonized during the embryonic stage, proliferate and differentiate under the influence of critical regulatory factors [2]. Among these, B cell activating factor (BAFF) and activation-induced cytidine deaminase (AID) are essential for the survival and selection of the antibody repertoire [3,4]. The development of the BF is a dynamic process, reaching a peak of activity during early growth, followed by physiological involution after sexual maturity [5,6]. The maturation and proliferation of B lymphocytes are observed in the medulla and at the cortico-medullary border, followed by an intense expansion of the the lymphoid follicle cortex. Consequently, the structural integrity of the BF, as determined by lymphocyte density and cortex/medulla ratio, directly correlates with the avian species’ capacity to counteract pathogenic challenges within intensive production systems [7,8].

In contemporary poultry farming, the optimization of environmental factors, particularly lighting, has emerged as a pivotal strategy to enhance growth performance and bird health [9]. Birds possess an exceptionally intricate visual system, exhibiting a spectral sensitivity that surpasses that of mammals [10,11]. This superior sensitivity is attributed to the presence of four distinct types of retinal cones, which facilitate the detection of different wavelengths of light. Additionally, the ability of light to penetrate the skull and stimulate hypothalamic photoreceptors further enhances the avian visual system’s sensitivity to light [12]. Recent research has demonstrated that short wavelengths have different effects on the physiology of broiler chickens. The green light (560 nm) has been observed to stimulate satellite cell proliferation and muscle development during the initial three weeks of life, thereby enhancing the meat quality and reducing oxidative stress [13,14,15,16]. The 480 nm blue light exhibited the greatest efficacy in the late growth phase (27–42 days old) in stimulating body weight and reducing aggression and stress [13,17,18]. Furthermore, the use of sequential light combinations, such as the transition from green to blue, demonstrated remarkable synergy, leading to more intense proliferation of peripheral T and B lymphocytes and a significant increase in IgG antibody titers against the Newcastle disease virus (NDV) [19,20].

The mechanism by which the light spectrum influences immunity is closely linked to the neuroendocrine activity of the pineal gland and melatonin secretion [21,22]. Melatonin, a hormone produced by the pineal gland, functions as a systemic mediator that translates external light signals into intracellular responses. It has the ability to regulate a number of physiological processes, including apoptosis, cell longevity, and mitochondrial function. This regulation occurs through the scavenging of free radicals and the stimulation of antioxidant enzymes [23,24,25]. It has been demonstrated that green light exerts a more pronounced effect on melatonin secretion in the pineal cells of chicks when compared with red or white light [26,27]. In birds, melatonin exerts its effects through three subtypes of membrane receptors (Mel1a, Mel1b, and Mel1c) [28,29,30] as well as through nuclear receptors in the ROR family [28,31]. A body of research has previously demonstrated that exposure to green light leads to an increase in plasma melatonin levels, a phenomenon that occurs as a result of the stimulation of pinealocytes. This stimulation, in turn, has been shown to enhance T-cell proliferation through the action of Mel1b and Mel1c receptors [32]. While the evidence suggests that melatonin modulates immunity and antioxidant capacity in chicks [16,33], the specific intracellular mechanisms by which it promotes B-cell proliferation in the BF under the influence of monochromatic light require further investigation.

The objective of this study was to address this knowledge gap by analyzing the impact of alternating green and blue wavelengths on the morphology of the BF and the mitotic activity of B lymphocytes in broiler chickens. The medullary area of the BF was chosen to calculate the lymphocyte density for three reasons. The first is that these B lymphocytes proliferate in both the cortex and the medulla, and only after 8–10 weeks does the number of lymphocytes inside the medulla decrease, indicating the onset of BF involution, a process that is completed at 6–7 months of age [5]. The second reason was that the medulla area represents the reserve and maturation compartment of B lymphocytes [34], and we monitored the evolution of cell occupancy in this critical space. The third reason was that the medulla area was easier to recognize and isolate as the area of interest for lymphocyte quantification.

The central hypothesis of this study posits that the implementation of an optimized light regimen over a period of six weeks results in the synthesis of melatonin, which acts on membrane receptors that are present within the BF, thereby activating intracellular signaling cascades that promote the clonal expansion of B lymphocytes. Furthermore, the evaluation of the growth performance was conducted to identify economical solutions that could enhance the immune performance of poultry. The present research investigates these interactions with the objective of elucidating the fundamental mechanisms of photoperiodic immunomodulation. In addition, it aims to provide practical solutions for the use of light-emitting diode (LED) technology to increase immune resistance and productive efficiency in sustainable poultry farming.

## 2. Materials and Methods

### 2.1. Animals and Experimental Design

A total of 336 one-day-old Ross 308 male and female broilers were used in this study. They were housed in the biobasis of the University of Life Sciences “Ion Ionescu de la Brad” in Iași, and were randomly assigned to four separate groups: WL-Male, G-GxB-BL-Male, WL-Female, and G-GxB-BL-Female. Each group was subdivided into 4 pens (*n* = 21 chicks) at a density of 11.5 birds/m^2^, and a total of 16 individual pens were included in the analysis as experimental units. To prevent accidental light exposure between adjacent cages, the cages were physically separated by opaque plastic partitions. Each cage was equipped with an LED strip emitting a specific wavelength, connected to a dimmer. The LED lights were installed at bird head level with an irradiance of 0.019 W/m^2^ and illuminance of 20 lux, and the light intensity was calibrated weekly (Table 1). The chicks in the WL-Male and WL-Female groups were exposed to white light (WL, 400–760 nm) for 6 weeks, while the chicks in the G-GxB-BL-Male and G-GxB-BL-Female groups were exposed to a combination of monochromatic lights as follows: green (560 nm) from 1 to 14 days of age, green and blue (480–560 nm) for 15–28 days of age, and blue lights (480 nm) for 29–42 days of age. In order to prevent light spillage, the two primary light treatments were installed in separate rooms. The lights were generated by a LED system provided by Greentek Lighting (DARA) (Bucharest, Romania) for six weeks.

The birds were reared under uniform environmental conditions in two specially designed rooms and were fed commercial diets (Table 2) in accordance with the animal welfare recommendations of the European Union Directive 86/609/EEC. The ambient temperature was initially maintained at approximately 32 °C for a period of three days. Thereafter, a reduction was made to 30 °C during the first week, followed by a further reduction to 28 °C in the second week. Subsequent to that, a decrease of 2–3 °C was implemented once a week. The relative humidity was maintained at 60–70% for the entire period. The feed was administered ad libitum, and the average daily feed intake (ADFI) was then calculated by subtracting the weight of the leftover feed from the total amount of feed provided. The feed conversion ratio (FCR) was calculated as the total feed consumed divided by the total body weight gain. The weekly mortality rate was calculated by dividing the number of birds that died during that week to the number of birds that were present at the beginning of the period. The uniformity of the flock was expressed as the coefficient of variation (CV%) of body weight for each specimen at weekly intervals throughout the growth cycle of the males and females in each poultry pen. This was calculated as the ratio of the standard deviation to the mean weight of a batch, multiplied by 100.

The experiments were conducted in accordance with the international ARRIVE guidelines 2.0 [35], with the approval of the Ethics Committee of the Faculty of Veterinary Medicine, Iași, within the University of Life Sciences “Ion Ionescu de la Brad” in Iași (nr. 1742/11 November 2024).

### 2.2. Experimental Measurements

After each week of life (i.e., at 7, 14, 21, 28, 35, and 42 days of age), 3 chicks from each replicate pen were selected. The process involved the simultaneous sampling of light-colored groups, with randomization of the order within each group. For each age group, a total of 24 male broilers and 24 female broilers were weighed, euthanized (cervical dislocation) and immediately dissected. Following dissection, the BF was collected and weighed. The evaluation of each bursa was conducted independently; however, the results were reported as a mean per cage. The experimental analyses were conducted in a blinded manner, as each cage was randomly numbered, and the researchers who performed the histological and immunohistochemical analyses were unaware of the grouping of the cages.

The bursa index is thus calculated as the ratio of the BF weight (in grams) to the chicken’s total body weight (in grams), multiplied by 100%.

#### 2.2.1. Histomorphometrical Analyses

##### Hematoxylin and Eosin Staining

The BF sample was divided into 2 parts for histological analysis and immunohistochemistry, respectively. The main procedures involved fixing the samples with 10% paraformaldehyde for at least 3 days, subsequently dehydrating them using an alcohol gradient, clarifying them with xylen and embedding them in paraffin wax. Sections with a thickness of 5 μm (microtom) were then stained with hematoxylin and eosin (H&E), and observed and photographed using a microscope (Microscop Leica, DM500, Wetzlar and Mannheim, Germany).

##### QuPath Analysis

In order to analyze and measure multiple morphometric indicators within the BF, 40× digital images were captured and QuPath software (version number 0.5.1) was utilized. These indicators included: the mean area of lymphoid follicles, the ratio between the cortical and medullary area, the lymphocyte density within the medulla of lymphoid follicles, and the thickness of the follicle-associated epithelium. In principle, three cross-sections were made from each BF, then five random fields were selected from in which the lymphoid follicles were measured. The areas of each lymphoid follicle and medulla were measured and expressed in mm^2^. The cortex area was derived by subtracting the medulla area from the total area of the lymphoid follicle. The lymphocyte density was measured exclusively in the medullary region of the lymphoid follicles by automatically counting the cells relative to the area of interest (1 × 10^4^ cells/mm^2^). Follicle-associated epithelial thickness was measured as the vertical distance from the innermost membrane to the outermost membrane of the epithelial cells.

#### 2.2.2. Immunohistochemical Analyses

Immunohistochemical analysis was performed by utilizing two distinct protocols for the three antibodies under investigation (Table 3), using the streptavidin–biotin–peroxidase method (RE7140K Kit, Novolink Polymer Detection System; Leica Biosystems, Newcastle, UK). For PCNA and Mel1a, following the procurement of histological sections from BF, the slides were subjected to a dewaxing process using xylene, followed by a series of ethyl alcohol baths for dehydration. Thereafter, the slides were immersed in citrate buffer at a temperature of 95 °C for a duration of 10 min, with a pH of 6. Subsequent to cooling, the slides underwent a washing step with citrate buffer, followed by an overnight incubation with the primary antibody in a humid chamber maintained at 4 °C. On the second day, the slides were washed with PBS and then incubated with the secondary antibody. For RORα, an EDTA pH of 9 at 100 °C was utilized. The washes were performed with TBS for one minute, and the primary antibody was left for only 45 min in a humid chamber. Subsequently, for chromogenic detection, all slides were subjected to 3,30-diaminobenzidine treatment, followed by contrast enhancement with hematoxylin. The slides were observed and photographed using a microscope. Digital images at 40× magnification were imported into QuPath using brightfield DAB modules. Cells identified as positive were automatically counted and expressed as the number of positive cells ×10^3^/mm^2^. The efficacy of this IHC analysis method is predicted based on its ability to determine the precise location of proteins in the tissue, in addition to the number of cells marked per unit of measurement.

### 2.3. Statistical Analysis

Statistical analysis was conducted using SPSS 22.0. A two-way ANOVA with repeated measures was employed to analyze the growth performance, morphometric indices and immunohistochemical data over the 6-week period. The model included lighting treatment (WL vs. G-GxB-BL) and sex (male vs. female) as between-subject factors, and time (days) as the repeated-measures factor. All interactions (light × sex, time × light, time × sex, time × light × sex) were evaluated. The pen (n = 4 per sex/light combination) was the experimental unit, with values from sampled birds being averaged per pen. The effect sizes were reported using partial eta squared (η^2^) to indicate the magnitude of the observed effects. The normality and homogeneity of variance were verified using Shapiro–Wilk and Levene’s tests, respectively. For repeated measures, Mauchly’s test of sphericity was applied. When significant effects were detected for light × sex, the means were compared using Tukey’s post hoc test. Data are expressed as mean ± standard error of the mean (SEM). Differences were considered to be statistically significant at *p* < 0.05.

## 3. Results

### 3.1. Effects of Combined Monochromatic Light on Growth Performance

To investigate possible changes induced by monochromatic lights on chick development, we measured and determined several indices such as body weight, ADG, ADFI, FCR, mortality, and flock uniformity (Table 4). The two-way repeated measures ANOVA confirmed significant effects of time (*p* < 0.001, η^2^ = 0.994), lighting treatment (*p* < 0.009, η^2^ = 0.450) and sex (*p* < 0.001, η^2^ = 0.927) on body weight. However, no significant interactions were found for Time × Light (*p* = 0.315, η^2^ = 0.092) or Light × Sex (*p* = 0.586, η^2^ = 0.025) suggesting that the growth-promoting effect of the G-GxB-BL lighting program was consistent across both sexes throughout the 6-week experimental period. Weekly univariate analyses (Table 4) revealed that the lighting treatment significantly influenced body weight at 28 days (*p* = 0.006, η^2^ = 0.479), with strong tendencies observed at 21 days (*p* = 0.074, η^2^ = 0.242) and 42 days (*p* = 0.091, η^2^ = 0.220). Although the *p*-values in the final weeks were slightly above the 0.05 threshold, the large effect sizes (η^2^ > 0.14) suggest a substantial biological impact of the G-GxB-BL throughout the growth period.

Regarding the ADG, the two-way repeated measures ANOVA confirmed significant effects of time (*p* < 0.001, η^2^ = 0.980), lighting treatment (*p* = 0.004, η^2^ = 0.520) and sex (*p* < 0.001, η^2^ = 0.937); however, no significant interactions were found for Light × Sex (*p* = 0.646, η^2^ = 0.018), suggesting that the growth-promoting effect of the lighting program was consistent across both sexes throughout the 6-week experimental period. The most pronounced effect was observed at 28 days, where birds in the G-GxB-BL group showed significantly higher weight gains (55.39 vs. 51.99 g/bird/day; *p* = 0.006, η^2^ = 0.479). Furthermore, strong positive tendencies were noted at 21 days (*p* = 0.074, η^2^ = 0.242) and at 42 days (*p* = 0.091, η^2^ = 0.220). The large effect sizes recorded from Week 3 onwards indicate that the G-GxB-BL light effectively stimulated protein deposition and growth, regardless of the birds’ sex.

Regarding the ADFI, the G-GxB-BL treatment led to a significant reduction in feed consumption compared to the WL group. This effect was highly significant at 7 days (*p* = 0.032, η^2^ = 0.328), 21 days (*p* = 0.005, η^2^ = 0.490) and 42 days (*p* = 0.008, η^2^ = 0.461). Throughout the experimental period, birds exposed to the G-GxB-BL spectrum consistently exhibited lower intake levels than the WL group, while maintaining higher growth rates. No significant Light × Sex interactions were observed (*p* = 0.387, η^2^ = 0.063), indicating that the lighting program (*p* < 0.001, η^2^ = 0.774) influenced feeding behavior similarly in both males and females (*p* < 0.001, η^2^ = 0.999) throughout the 6-week experimental period (*p* < 0.001, η^2^ = 0.999).

The FCR was significantly improved by the G-GxB-BL treatment. The two-way repeated measures ANOVA confirmed significant effects of time (*p* < 0.001, η^2^ = 0.956), lighting treatment (*p* = 0.007, η^2^ = 0.468) and sex (*p* = 0.002, η^2^ = 0.581) on the feed conversion ratio. The most substantial enhancement in feed efficiency was recorded at 28 days (*p* = 0.008, η^2^ = 0.459) and at 21 days old (*p* = 0.034, η^2^ = 0.323). A strong statistical tendency toward better FCR was also observed in the final week of the trial (42 days, *p* = 0.059, η^2^ = 0.267). No significant interactions between light and sex were found (*p* = 0.452, η^2^ = 0.048), indicating that the G-GxB-BL spectrum optimized nutrient utilization efficiency in both males and females.

The mortality rate remained very low throughout the experimental period, with no significant differences observed between lighting programs or sexes at 7 days old (*p* = 0.175, η^2^ = 0.148). Remarkably, from 28 days to the end of the trial, no mortality was recorded in any of the experimental groups (0.00%). These results indicate that the G-GxB-BL lighting schedule did not negatively affect the birds’ viability or health status.

Regarding flock uniformity, a significant effect from the lighting program was observed at 14 days old (*p* = 0.044, η^2^ = 0.296), where birds under the G-GxB-BL lighting schedule showed superior weight consistency. For the remainder of the trial (21–42 days), no significant differences were detected between the lighting treatments (*p* = 0.366, η^2^ = 0.069) or sexes (*p* = 0.389, η^2^ = 0.062), indicating that the G-GxB-BL lighting schedule maintained a stable and uniform growth pattern across the entire flock, regardless of the birds’ sex (P-L × S = 0.784, η^2^ = 0.007).

### 3.2. Effects of Combined Monochromatic Light on the Development of BF

**Figure 1:**
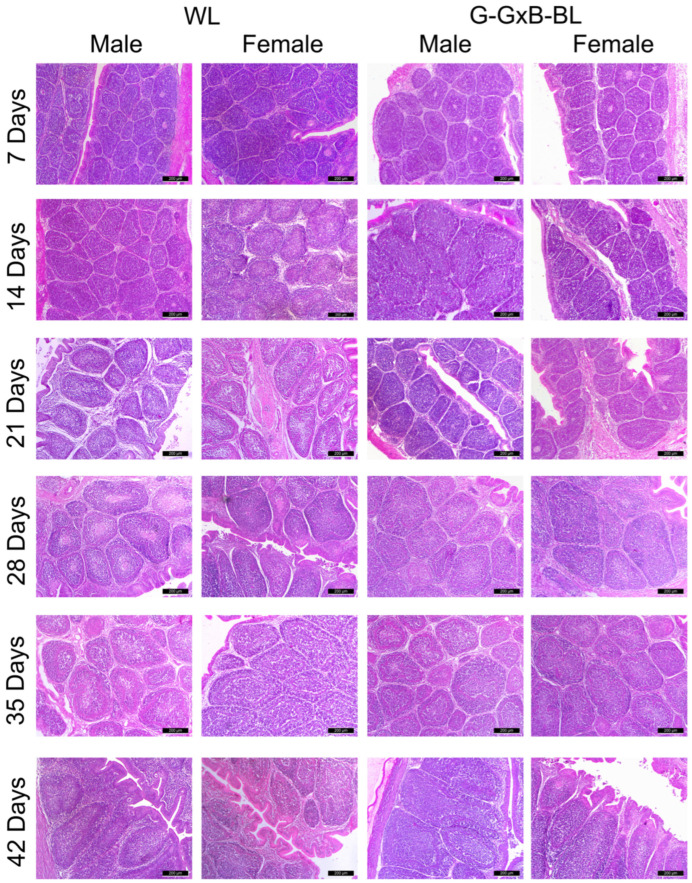
The impact of the combination of green and blue monochromatic lights on the development of the BF in male and female broiler chickens aged between 7 and 42 days. The morphological structure of the BF was similar at each time point for both groups, with no obvious abnormalities. The bursal wall of broilers comprised three layers from the outside to inside: Tunica serosa, Tunica muscularis and Tunica mucosa. The bursal mucosa consists of numerous longitudinal folds known as the bursal plicae, which contain lymphoid follicles, lamina propria, and two types of epithelia. The center of the plicae consists of highly vascularized connective tissue from which small septa formed of reticulin and collagen fibers will detach, surrounding each lymphoid follicle with oval or irregular shapes. Starting at 7 days of age, a clear distinction can be observed between the cortex and medulla zones of the lymphoid follicles, as numerous lymphocytes begin to accumulate in the cortex, making it appear more compact than the loose medulla zone. Abbreviations: WL: white light and G-GxB-BL: green and blue light. H&E stain. Scale bar: 200 µm.

**Figure 2:**
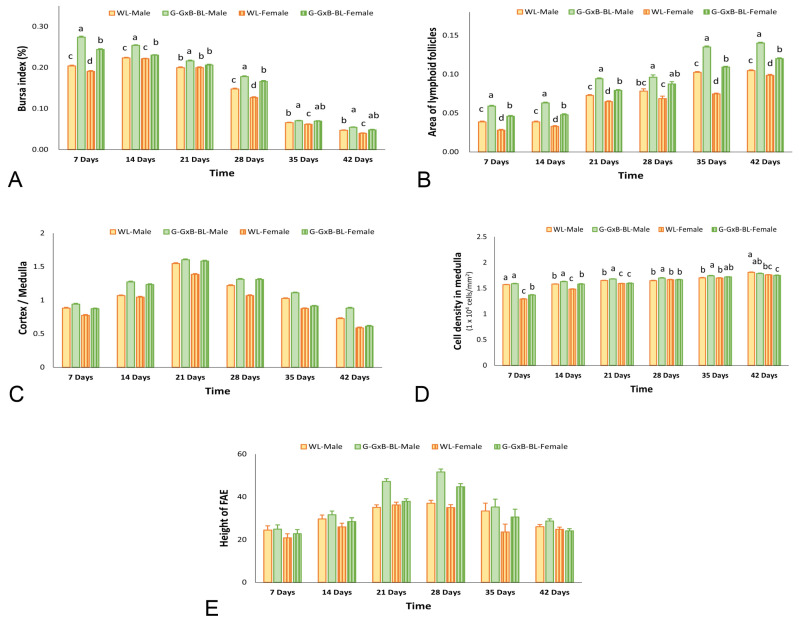
Graphical representation of the impact of the combination of green and blue monochromatic lights on the development of the BF in male and female broiler chickens aged between 7 and 42 days. (**A**) Bursa index, (**B**) aria of lymphoid follicles, (**C**) cortex/medulla ratio, (**D**) lymphocyte density in the medulla, and (**E**) height of FAE. Data are presented as means ± SEM. ^a,b,c,d^ The presence of different superscripts within the same week indicates significant differences (*p* < 0.05) based on Tukey’s HSD test, subsequent to the observation of a Light × Sex interaction.

This study further explores the effects of mixing monochromatic green and blue lights on the immune response of chickens by systematically evaluating its impact on the morphology of the BF using H&E staining (Figure 1). Throughout the entire experimental period, for both types of light, the stock index values followed a trend within the physiological limits, peaking in the first weeks of life and then declining until day 42. Regarding the organ index, a significant Light × Sex interaction was observed (*p* = 0.012, η^2^ = 0.418), indicating that the effect of the monochromatic light spectrum was sex-dependent (Supplementary Table S1). Furthermore, the triple interaction (Time × Light × Sex) reached statistical significance (*p* = 0.018, η^2^ = 0.254), suggesting that the combined influence of lighting and sex on organ development fluctuated throughout the 6-week period. Our results demonstrate that exposure to G-GxB-BL significantly increased (*p* < 0.05) the bursa index in broiler chickens compared to the WL group in all age categories evaluated (Figure 2A).

The lymphoid follicles, which represented the functional units of the BF, increased from days 7 to 42 days of age. Regarding the aria of lymphoid follicles, the two-way repeated measures ANOVA revealed highly significant main effects for time, light, and sex (*p* < 0.001). Mauchly’s test indicated a violation of sphericity (*p* = 0.001); thus; the Greenhouse–Geisser correction was applied. A strong significant Light × Sex interaction was observed (*p* = 0.003, η^2^ = 0.545), indicating that the effect of the light treatment spectrum was sex-dependent. Most notably, the triple interaction of Time × Light × Sex was highly significant (*p* = 0.010, η^2^ = 0.345), suggesting that the combined influence of the lighting program and sex on the aria of lymphoid follicles followed distinct trajectories throughout the 6-week experimental period. Quantitative analyses indicated that broilers exposed to G-GxB-BL lights exhibited significantly larger lymphoid follicles areas compared to the WL group (*p* < 0.05) (Figure 2B).

The ratio between the cortical and medulla zones has been shown to reflect the immune maturation of BF. It has been demonstrated that the BF evolves from a phase of cortical organization at 7 days to a functional and structural peak at 21 days, followed by a decrease in the values due to cortical thinning and physiological regression of the BF. The two-way repeated measures ANOVA revealed a highly significant effect of time (*p* < 0.001, η^2^ = 0.998), light (*p* < 0.001, η^2^ = 0.951) and sex (*p* = 0.001, η^2^ = 0.943) on the cortex and medulla ratio. No significant interaction was found between Light × Sex (*p* = 0.202, η^2^ = 0.136), suggesting a consistent response to the light program across both sexes. While time significantly influenced the cortex and medulla ratio, its interactions with light and sex showed a significant effect (*p* = 0.000, η^2^ = 0.786) and, respectively (*p* = 0.000, η^2^ = 0.847), suggests that the rate at which the ratio between the cortical and medullary zones changed differed among groups as the chicks grew (Figure 2C). The cortex and medulla comprised a population of cells that are distributed randomly. These cells include numerous lymphoblasts, lymphocytes, reticular cells, macrophage cells, and dendritic and plasma cells. The boundaries of these cells could not be demonstrated under the light microscope; therefore, their identification was based on the presence of nuclei. The nuclei of the lymphoblasts were characterized by substantial size and clear configuration, with one or two prominent nucleoli. The lymphocytes were distinguished by the presence of dark heterochromatic nuclei. The reticular cells were characterized by the presence of large, light oval-shaped nuclei. The nucleus of macrophages manifested an ovoid shape. The nuclei of the dendritic cells were predominantly a pear-to-elliptical shape, and were characterized by the presence of peripheral heterochromatin, accompanied by a condensed nucleolus. The nuclei of plasma cells manifested a cartwheel-like configuration. Knowing the cell population and morphology of the lymphocyte nucleus, we calculated the lymphocyte density in the medullary area. The two-way repeated measures ANOVA revealed highly significant main effects for time, light, and sex (*p* < 0.001) on the density of lymphocytes in the medullar zone. A strong significant Light × Sex interaction was observed (*p* = 0.008, η^2^ = 0.458), indicating that the effect of the G-Gxb-BL spectrum was sex-dependent. Most notably, the triple interaction of Time × Light × Sex was highly significant (*p* < 0.001, η^2^ = 0.575), suggesting that the combined influence of the lighting program and sex on the density of lymphocytes in the medullar zone followed distinct trajectories throughout the 6-week experimental period. The use of G-GxB-BL resulted in significantly higher lymphocyte densities (*p* < 0.05) compared to WL in females at 7 days old, males and females at 14 days old and males at 21–28–35 days old (Figure 2D).

Bursal plicae was covered by two types of surface epithelium—IFE (inter follicular epithelium) and FAE (follicle-associated epithelium)—which provide a direct connection between the bursal lumen and the follicular medulla. FAE includes: columnar cells with nuclei at different heights, in most cases found apically; M cells with clear cytoplasm, which are involved in antigen transport; and intraepithelial and intraluminal lymphocytes with dark, rounded nuclei. The height of the FAE epithelium commenced at a low value in the initial week, which was attributable to the presence of cubic–columnar cells. These cells underwent a gradual increase until they attained structural maturity at 21 and 35 days of age, exhibiting the maximum height. Thereafter, the cells underwent a gradual return to their cubic–columnar configuration towards the end of the experimental period (Figure 1). The two-way repeated measures ANOVA revealed highly significant main effects for the lighting treatment (*p* < 0.001, η^2^ = 0.679) and sex (*p* = 0.001, η^2^ = 0.631) on the FAE height. A significant Time × Light interaction was detected (*p* = 0.001, η^2^ = 0.287), indicating that the growth rate of the FAE height was significantly modulated by the light treatment throughout the experimental period. No significant interactions were found between Time × Sex (*p* = 0.645), Time × Light × Sex (*p* = 0.100) or Light × Sex (*p* = 0.312), suggesting that the lighting program’s influence on FAE height followed a similar developmental trajectory in both males and females (Figure 2E).

### 3.3. Impact of Mixing Monochromatic Light Exposures on PCNA, Mel1a and RORα Expression in Cell of BF

**Figure 3:**
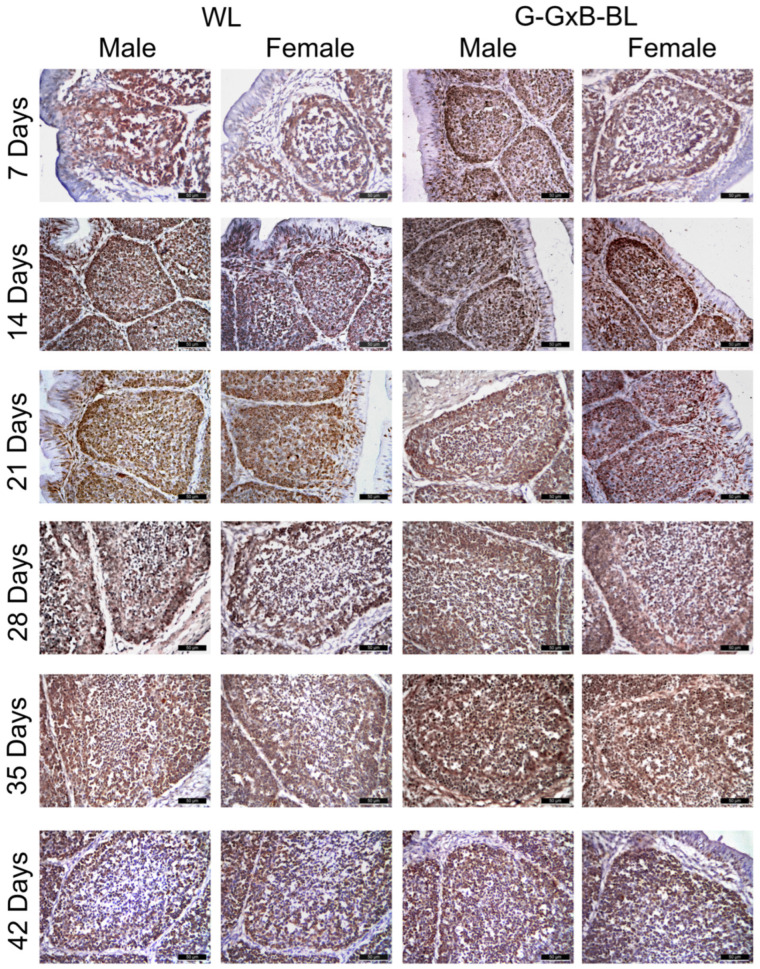
Immunohistochemical analysis of PCNA in the BF due to the impact of the combination of green and blue monochromatic lights on male and female broiler chickens aged between 7 and 42 days. PCNA-positive cells were distributed in both the cortical and medullary regions of the BF. At 7–14 days of age, PCNA expression was intense, as nearly all B lymphocytes are in a phase of active cellular division and proliferation to populate the lymphoid follicles. The highest levels of PCNA immunolabeling were recorded at 21 and 28 days of life, with the cortex being particularly notable for its advanced development at these ages. At 35 and 42 days of life, a decrease in PCNA immunomarking was observed in lymphoid follicles, reflecting the onset of the physiological involution of BF. Exposure to G-GxB-BL resulted in a significant increase in the cell renewal rate, as indicated by a notable rise in the number of PCNA-positive cells in G-GxB-BL groups when compared to the WL groups. Scale bar is 50 μm.

**Figure 4:**
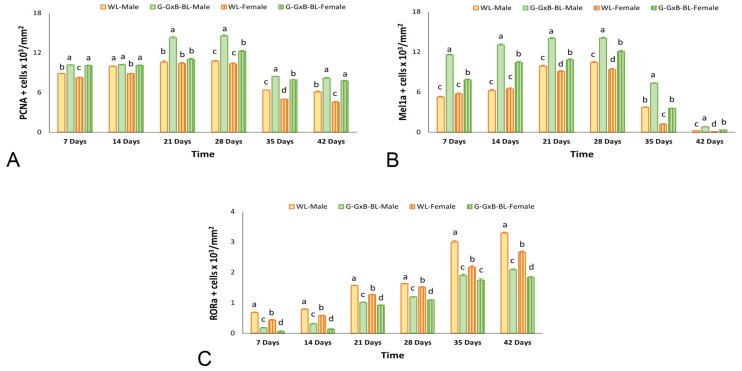
Graphical representation of immunohistochemical analysis for the antibodies PCNA, Mel1a, and RORα, due to the impact of the combination of green and blue monochromatic lights on the development of BF in male and female broiler chickens aged between 7 and 42 days. (**A**) Positive PCNA cell number, (**B**) positive Mel1a cell number, and (**C**) positive RORα cell number. Data are presented as means ± SEM. ^a,b,c,d^ The presence of different superscripts within the same week indicates significant differences (*p* < 0.05) based on Tukey’s HSD test, subsequent to the observation of a Light × Sex interaction.

**Figure 7:**
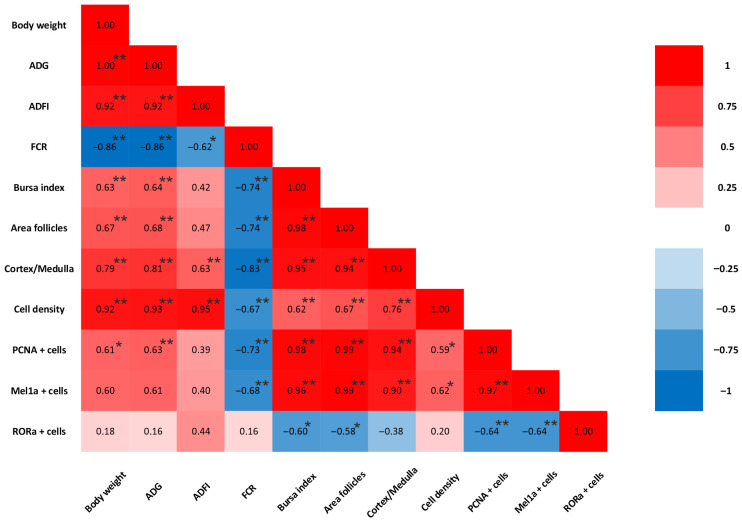
Heatmap graph of Spearman’s correlation coefficient (r). A strong positive correlation is colored red, a strong negative correlation is colored blue, and a weak correlation is colored white. (*) Correlation is significant at the 0.05 level. (**) Correlation is significant at the 0.01 level. Heat map of Pearson’s correlation coefficient (R).

The proliferation activity of bursa cells in 7- to 42-day-old broiler chickens was assessed using IHC staining. PCNA-positive cells exhibited brown nuclei that were distributed in both the cortical and medullary regions of the BF (Figure 3). At 7–14 days of age, PCNA expression was intense, as nearly all B lymphocytes are in a phase of active cellular division and proliferation to populate the lymphoid follicles. Regarding PCNA-positive cells, the two-way repeated measures ANOVA revealed highly significant main effects for time, lighting treatment, and sex (*p* < 0.001), with large effect sizes (η^2^ > 0.984). A significant Light × Sex interaction was found (*p* = 0.011, η^2^ = 0.427), alongside a highly significant triple interaction of Time × Light × Sex (*p* < 0.001, η^2^ = 0.864) (Supplementary Table S2). These results indicate that the G-GxB-BL promoted the number of PCNA-positives cells through distinct trajectories for males and females over the 6-week experimental period. A quantitative assessment (Figure 4A) showed that exposure to a mixed green and blue monochromatic lights significantly increased the density of PCNA-positive cells in the BF in G-GxB-BL-Male at 7–21–28–35 and 42 days of age compared to WL-Male (*p* < 0.05). Furthermore, in G-GxB-B-Female chickens, a similar increase was observed at 7–14–28–35 and 42 days of age (*p* < 0.05), in comparison to WL-Female. The highest levels of PCNA immunolabeling were recorded at ages 21 and 28 days of life, with the cortex being particularly notable for its advanced development at these ages. Exposure to G-GxB-BL resulted in a significant increase in the cell renewal rate, as indicated by a notable rise in the number of PCNA-positive cells in G-GxB-BL-Male (14.25 ± 0.17, respectively 14.55 ± 0.12) when compared to the WL-Male group (10.60 ± 0.17, respectively 10.75 ± 0.12). At 35 and 42 days of life, a decrease in PCNA immunomarking was observed in lymphoid follicles, reflecting the onset of the physiological involution of BF. In addition, Spearman’s correlation analysis showed that the number of PCNA-positive cells is positively correlated with the bursa index (r = 0.98, *p* < 0.001), the area of lymphoid follicles (r = 0.99, *p* < 0.001), the ratio between the cortex and medulla of lymphoid follicles (r = 0.94, *p* < 0.001), the lymphocytes density (r = 0.59, *p* = 0.015) and the number of Mel1a + cells (r = 0.97, *p* < 0.001) and is negatively correlated with the number of RORa + cells (r = −0.64, *p* = 0.008) (Figure 7).

**Figure 5:**
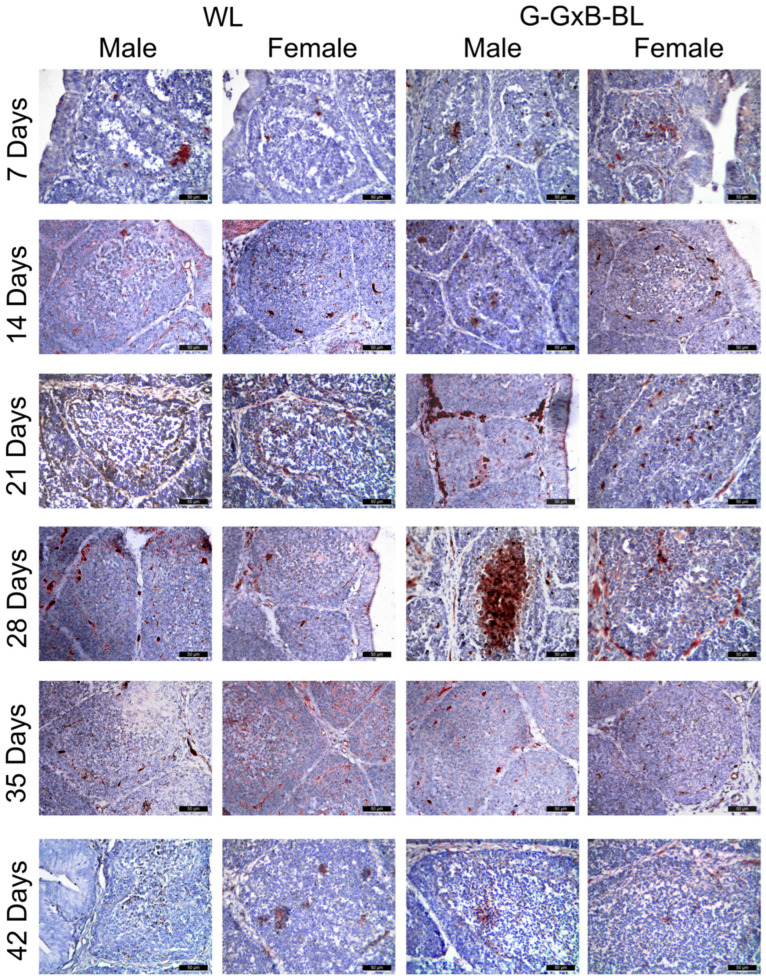
Immunohistochemical analysis of Mel1a in the BF due to the impact of the combination of green and blue monochromatic lights on male and female broiler chickens aged between 7 and 42 days. Mel1a-positive cells were predominantly distributed within the medulla, specifically at the cortico-medullary junction and the vascular walls. While Mel1a expression was moderate in maturing B lymphocytes between 7 and 14 days of age, it reached peak intensity at 21 and 28 days, aligning with the period of maximum immunological activity in the bursa of Fabricius. Furthermore, the application of mixed green and blue monochromatic lights (G-GxB-BL) significantly increased the number of Mel1a+ cells in both the male and female groups across all ages compared to the white light (WL). As the gland undergoes involution, there is a concomitant decrease in the number of melatonin receptors, which results in a marked reduction in the number of Mel1a-positive cells in the lymphoid follicles. Scale bar is 50 μm.

In order to demonstrate its immunostimulatory activity and the link between the immune and neuroendocrine systems in BF, an IHC assessment of Mel1a was conducted in broiler chickens for six weeks (Figure 5). The distribution of positive cells for Mel1a was predominantly located in the medulla, specifically at the cortico-medullary junction and within the vascular wall. For a period of 7–14 days, the expression of Mel1a is moderate and is present in maturing B lymphocytes. Regarding Mel1a-positive cells, the two-way repeated measures ANOVA revealed highly significant main effects for time, lighting treatment, and sex (*p* < 0.001), with large effect sizes (η^2^ > 0.980). A significant Light × Sex interaction was found (*p* < 0.001, η^2^ = 0.949), alongside a highly significant triple interaction of Time × Light × Sex (*p* < 0.001, η^2^ = 0.995). These results indicate that the G-GxB-BL increased the number of Mel1a-positive cells through distinct trajectories for males and females over the 6-week experimental period. The utilization of a mixture of green and blue monochromatic lights resulted in a substantial augmentation in the number of Mel1a + cells in the G-GxB-BL-Male and the G-GxB-BL-Female, in comparison to the WL-Male and WL-Female for all age groups (*p* < 0.05). Immunolabeling was found to be more intense at 21 and 28 days of age (14.00 ± 0.13, respectively 14.06 ± 0.16), which coincides with the period of maximum immunological activity of the BF. As the gland involutes, the number of melatonin receptors decreases, resulting in a marked reduction in the number of Mel1a-positive cells in the lymphoid follicles. However, the utilization of G-GxB-BL lights resulted in an augmentation of the number of Mel1a-positive cells at 35 and 42 days of age in both G-GxB-BL-Male and G-GxB-BL-Female in comparison to WL-Male and WL-Female (Figure 4B). In addition, Spearman’s correlation analysis showed that the number of Mel1a-positive cells is positively correlated with the bursa index (r = 0.96, *p* < 0.001), the area of lymphoid follicles (r = 0.99, *p* < 0.001), the ratio between the cortex and medulla of lymphoid follicles (r = 0.90, *p* < 0.001), the lymphocytes’ density (r = 0.62, *p* = 0.010), and is the number of PCNA + cells (r = 0.97, *p* < 0.001), and is negatively correlated with the number of RORa + cells (r = −0.64, *p* = 0.008) (Figure 7).

**Figure 6:**
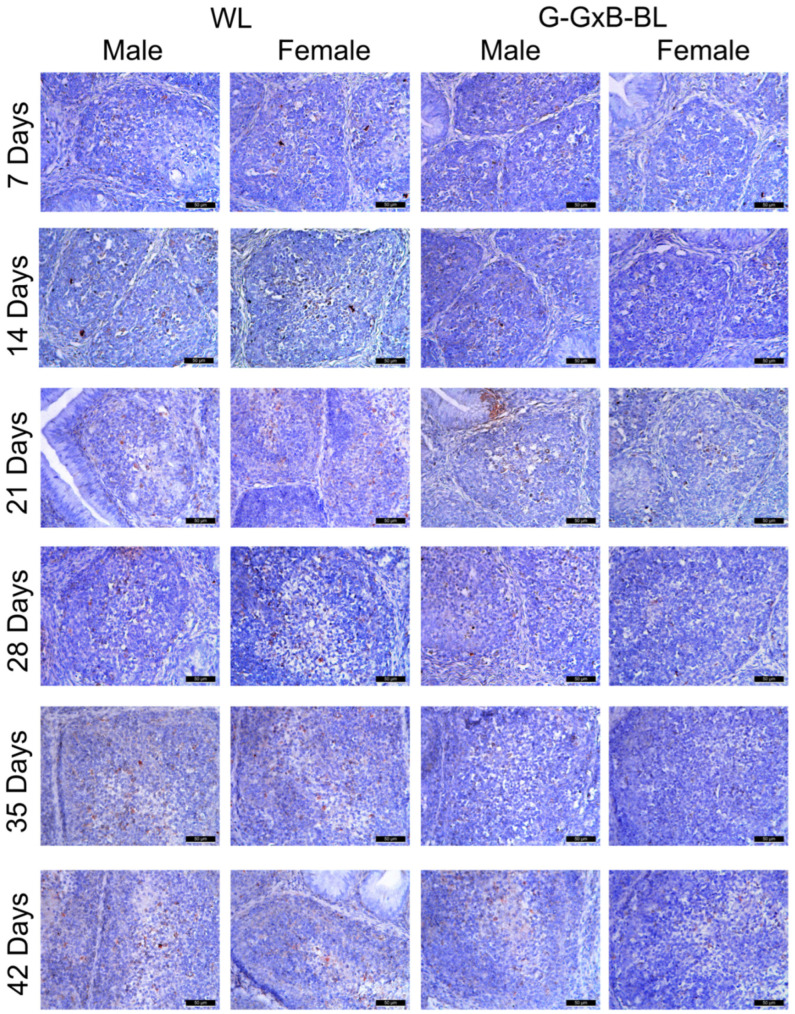
Immunohistochemical analysis of RORα in the BF due to the impact of the combination of green and blue monochromatic lights on male and female broiler chickens aged between 7 and 42 days. RORα-positive cells were distributed in both the cortex and medulla of lymphoid follicles. At 7–14 days of age, RORα expression was weak, as those B lymphocytes that had not passed the initial stages of proliferation and differentiation in the medulla were labeled. The highest number of RORα-positive cells was calculated at the oldest ages, when the BF was preparing for its physiological involution and for the gradual loss of B lymphocytes. Immunohistochemical analysis revealed that exposure of chicks of both sexes to a mixture of green and blue light (G-GxB-BL) significantly reduced the density of RORα-positive cells in the bursa of Fabricius, indicating a decrease in melatonin receptor-mediated apoptosis. This decline in the RORα marker, observed up to 42 days of age, indicates that the monochromatic lighting regimen delays the physiological involution of the organ and supports the immune activity of B lymphocytes. Scale bar is 50 μm.

In order to demonstrate the potential involvement of the nuclear receptor for melatonin in the processes of apoptosis and the reduced immune activity of B lymphocytes in BF, an immunohistochemistry (IHC) staining procedure was initiated. RORα-positive cells were distributed in both the cortex and medulla of lymphoid follicles (Figure 6). At 7–14 days of age, RORα expression was weak, as those B lymphocytes that had not passed the initial stages of proliferation and differentiation in the medulla were labeled. A quantitative assessment (Figure 5) demonstrated that two-way repeated measures ANOVA revealed highly significant main effects for time, lighting treatment, and sex (*p* < 0.001), with large effect sizes (η^2^ > 0.970). A significant Light × Sex interaction was found (*p* < 0.001, η^2^ = 0.892), alongside a highly significant triple interaction Time × Light × Sex (*p* < 0.001, η^2^ = 0.709). These results indicate that the G-GxB-BL modulated the number of RORa-positives cells through distinct trajectories for males and females over the 6-week experimental period. The exposure to a mixture of green and blue lights significantly diminished the density of RORα-positive cells in the G-GxB-BL-Male and G-GxB-BL-Female groups for all age groups (*p* < 0.05) in comparison to the WL-Male and G-GxB-BL-Female groups (Figure 4C). The highest number of RORα-positive cells was calculated at the oldest ages, when the BF was preparing for its physiological involution and for the gradual loss of B lymphocytes, which either migrated to secondary lymphoid organs or underwent apoptosis. This finding indicates that G-GxB-BL enhances the immune activity of B lymphocytes, and, by extension, that of BF, by reducing the degree of apoptosis in lymphoid follicles, both in male and female broilers. In addition, Spearman’s correlation analysis showed that the number of RORα-positive cells is negatively correlated with the bursa index (r = −0.60, *p* = 0.014), the area of lymphoid follicles (r = −0.58, *p* = 0.018), the number of PCNA + cells (r = −0.64, *p* = 0.008) and the number of Mel1a + cells (r = −0.64, *p* = 0.008) (Figure 7).

## 4. Discussion

This study shows that, compared to monochromatic white light, changing the ambient light spectrum from green to a combination of green and blue at 14 days of age, followed by another change to monochromatic blue light at 28 days of age, improved the feed conversion ratio and supported optimal development of the lymphoid system of bursa in broiler chickens. The maintenance of consistent farming conditions and the feeding program indicate that the light color is a significant factor in the growth of animals. The use of the G-GxB-BL monochromatic light combination optimized body weight at 28 days (*p* = 0.006, η^2^ = 0.479), with strong tendencies observed at 21 days (*p* = 0.074, η^2^ = 0.242) and 42 days (*p* = 0.091, η^2^ = 0.220). The lighting treatment also significantly influenced feed utilization at 28 days (*p* = 0.008, η^2^ = 0.459) and at 21 days old (*p* = 0.034, η^2^ = 0.323) and a strong statistical tendency toward better FCR was also observed in the final week of the trial (42 days, *p* = 0.059, η^2^ = 0.267). A number of studies have reported a positive effect of green and blue light on body weight, with this effect being most evident at the early stage [36,37,38]. As indicated by the findings of previous research, the body weight of broilers was observed to be elevated under green light and blue light in comparison to WL from 5 to 21 days [17] and from 2 to 6 weeks [39], with a greater increase in weight recorded at 2–5 weeks of age [37]. Furthermore, in addition to the benefits of using monochromatic lights, recent studies have indicated that the combination of the advantageous properties of monochromatic light has a significant impact on the growth patterns of broilers [38,40,41]. The final weight of the birds was found to increase by 10.66% in the presence of a green and blue mixed LED light in comparison with white light [42].

Furthermore, it has been demonstrated that light can also affect non-visual brain responses, which in turn impact behavior and emotional states. It is evident that a variety of wavelengths possess the capacity to activate non-retinal photoreceptors. As posited by Hartwig and van Veen [43], light comprising long wavelengths is more efficacious in traversing the skull and attaining non-retinal photoreceptors. Conversely, light of short wavelength necessitates higher intensity to activate non-retinal photoreceptors in the hypothalamus [44]. Furthermore, the wavelength of the light may also have an impact on melatonin levels and circadian rhythms [45], and these can in turn correlate with changes in behavior and affective states [46]. This specificity has the potential to provide a novel explanation for why different wavelength treatments affect bird behavior, if similar processes are indeed observed. Nevertheless, the mechanisms underlying these behavioral changes remain to be fully elucidated. This underscores the necessity for further research to comprehend the underlying causes of changes in behavior, fear and stress levels. The present experiment involved the separation of broiler chickens according to their sex, with the objective of ascertaining whether the combination of green and blue monochromatic lights exerted divergent influences on the CV for males and females. In the context of the present study, it was found that G-GxB-BL lighting schedule maintained a stable and uniform growth pattern across the entire flock, regardless of the birds’ sex (P-L × S = 0.784, η^2^ = 0.007). However, the present findings align with those of [47], who reported that the light color did not affect flock uniformity at 14 or 28 days of age. Further investigations are required in this regard, which may be combined with behavioral observations.

A series of studies have shown that the development of BF is influenced by several internal factors such as endocrine glands and sex hormones [48], or internal factors such as temperature [49], photoperiod [16,50], and minerals in the diet [51]. It is widely acknowledged that the organ index and morphometric analysis of the total lymphoid follicle surface area are associated with the degree of BF development [52] and with increased immune organ index or histological changes. This suggests that improved immune function and increased ability to resist various infections, diseases, and stress may be evident [53,54]. Depending on the photoperiod and its role in regulating hormones, body weight and tissue weight may change with the age of the bird [55]. Research has demonstrated that a reduced photoperiod can yield a favorable outcome, manifesting as an enhanced immune response in broiler chickens. This phenomenon may be attributable to the synthesis and secretion of melatonin [56]. Conversely, exposure to a suboptimal photoperiod has been demonstrated to induce a stress response in broilers, characterised by an increase in plasma corticosterone levels [57]. Indeed, experimental evidence has demonstrated that plasma corticosterone, induced under controlled conditions, has a demonstrable impact on the weight of primary and secondary lymphoid organs in chicks and on the reduction in the response in mitogens by reducing B and T cell proliferation [58]. Nevertheless, Nuthalapati et al. [59] demonstrated that varying degrees of photoperiod did not result in a reduction in the bursa index and did not cause significant levels of stress in the broiler chickens.

A number of studies have demonstrated the effectiveness of different light colors and photoperiod regimes on immune responses in birds [60,61]. However, research into the impact of combined colored LED lights on bird immune responses remains inconclusive. In the present experiment, we observed that G-GxB-BL light, compared to WL, favored a significantly higher organ index for the BF and determined a faster morphological development of lymphoid follicles by significantly increasing the area and density of lymphocytes within them. These results support our well-established role of light signaling (P-L × S = 0.012, η^2^ = 0.418), particularly green and blue light, in promoting immune organ development [62]. Although this research is limited by the fact that we did not measure the plasma melatonin levels, we believe that G-GxB-BL light may modulate melatonin activity and secretion, which can lead to improved morphological development of the BF and overall immune activity in broiler chickens. Similar results were obtained by Xie et al. [18], who demonstrated that cellular and humoral immune responses in broiler chickens are improved by green and blue LEDs. Additionally, Li et al. [16] proposed that green LED illumination could enhance the antioxidant capacity and melatonin secretion to induce B lymphocyte proliferation in the BF of young broilers. Recently, Cheng et al. [50] reported that exposing Yangzhou geese to green and blue light increased the index of the bursa organ and expanded the morphological area of bursal lymphoid follicles.

To further explain our hypothesis that the G-GxB-BL lights can have a positive effect on the immune system, we correlated the histomorphometric results with immunohistochemical staining of PCNA, which is a marker for evaluating the state of cell proliferation. Our study found that the number of PCNA-positive cells was significantly higher in the BF of broilers when exposed to G-GxB-BL versus WL in all age groups (*p* < 0.05). These data indicated that light-induced changes in PCNA immunostaining in the BF were correlated with increases in the bursa index, the mean area of lymphoid follicles, and the lymphocyte density. Consequently, our results suggest that B-cell proliferation may be enhanced by exposure to green light during the initial growth phase, to a combination of green and blue light during the final growth phase, and to blue light during the finishing phase, with results that are superior to those obtained with white light. This finding aligns with that of Xie et al. [18], who found that green light may extend the duration of antibody effectiveness and stimulate antibody production during the initial posthatching stage in broilers. Additionally, a combination of green and blue monochromatic light may enhance the mitogenic activity and B-lymphocyte proliferation, thereby increasing B-cell antibody production.

However, the mechanism by which these external light signals affect the lymphocyte proliferation in the BF remains unclear. Melatonin is a hormone that is secreted by the pineal gland primarily during periods of nocturnal rest, and its secretion may be influenced by environmental light stimuli. The application of monochromatic green light has been demonstrated to induce the expression of arylalkylamine N-acetyltransferase (AANAT) mRNA in chick pinealocytes and retinas, as well as to stimulate the secretion of melatonin [26]. According to the existent literature, melatonin-mediated green light has been shown to inhibit thymus T lymphocyte apoptosis [63] and increase T and B lymphocyte proliferation in the spleen [64]. Moreover, an exogenous melatonin supplement has been demonstrated to inhibit the development and maturation of mouse bone marrow B lymphocytes [65].

Nevertheless, the findings indicated that light-induced modification in PCNA immunolabeling and lymphocyte proliferation within the BF exhibited concordance with the changes in Mel1a immunolabeling in broilers. Consequently, we hypothesize that light signaling may regulate immune organ development through the modulation of melatonin receptors. Melatonin receptors have been shown to exhibit a tissue-specific distribution and expression profile, thereby facilitating melatonin’s ability to mediate a variety of physiological effects via distinct receptor subtypes [66,67]. Three membrane receptors—Mel1a, Mel1b and Mel1c—exist in avian species [68] and are expressed differently in BF under various light treatments. The Mel1a and Mle1c melatonin receptors are more important subtypes in melatonin-induced B lymphocyte proliferation than Mel1b in BF [29,69] and Mel1a immunolabeling was principally located in the medulla and cortico-medullary junction [29]. In the present study, we found the same wider distribution of Mel1a imunohistochemical staining in BF; more than that, we also found out that the number of Mel1a-positive cells was significantly higher in the BF of broilers when exposed to G-GxB-BL versus WL in all age groups. These results were initially confirmed by Li et al. [29], who concluded that exposure to green light increased melatonin secretion and the binding to Mel1a, which inhibits cAMP/PKA signaling via Gi proteins and the enhancement of B lymphocytes proliferation. A few years later, Zhang et al. [69] confirmed all of this and explicated that green and blue light illumination leads to a direct increase in melatonin secretion, which in turn promotes B-lymphocyte proliferation by binding to the melatonin receptor (Mel1a), which activates the Gi/PI3K/AKT signal. It has been demonstrated that, over the course of time, the AKT/GSK-3β/β-catenin signaling pathway is indispensable for the survival and development of B lymphocytes within the BF [70].

Melatonin, a compound that exhibits amphiphilic properties, has demonstrated a high degree of permeability across biological membranes. In addition to its membrane-bound receptors, studies have discovered that melatonin activates the nuclear receptors of retinoic acid-related orphan receptor a (RORα), RORβ, and RORγ [71], and the existence of a membrane–nuclear signaling pathway has been demonstrated [72]. There are studies showing that RORα and RORγ are mainly involved in the regulation of the immune system, while RORβ is mainly found in the nervous system [73,74]. The Pearson’s correction results demonstrated a robust negative correlation between the RORα or RORγ mRNA level and plasma melatonin concentration, thereby suggesting that melatonin impedes B lymphocyte apoptosis by negatively regulating the expression of the nuclear receptors RORα or RORγ [75]. Our Pearson’s correction results demonstrated showed that the number of RORα-positive cells is negatively correlated with the number of Mel1a + cells (r = −0.64, *p* = 0.008). As demonstrated by Zhao et al., the regulation of RORα expression by melatonin is dose-dependent. In other words, an increase in the exogenous melatonin concentration results in a decrease in RORα mRNA expression [76]. Furthermore, Wang et al. [77] demonstrated that melatonin can effectively reduce the mRNA and protein expression levels of RORγ. In this study, we examined the immunohistochemistry of RORα in the BF and found that positive cells were distributed in both the cortex and medulla of the lymphoid follicles. Furthermore, we found that the number of RORα-positive cells was significantly lower in the BF of broiler chickens exposed to G-GxB-BL compared to WL during the first four weeks of life. Similarly to our findings, there are studies that have identified that the combination of green and blue lights reduced the RORα mRNA level in the BF compared with white lights in chicken [75]. The same results were obtained in other lymphoid organs like thymus, where melatonin increased T lymphocyte proliferation by negatively regulating RORα expression under monochromatic green light [64].

After using G-GxB-BL lights, we obtained the following results: more efficient growth performance through a significant decrease in FCR and a more active immune function in the BF, as demonstrated by morphohistometry and immunohistometry. This light composition used in the chick rearing system from one day to 42 days of age resulted in an increase in the organ index. Although the BF index values generally showed a progressive decrease, this phenomenon reflected a physiological allometric growth in which body mass development exceeded the growth rate of the BF. This hypothesis is supported by the continuous growth of the follicular area, which demonstrated a structural expansion of the lymphoid tissue by the end of the experimental period. G-GxB-BL lights favored an increase in the follicular area, where a higher lymphocyte density was observed, as well as an increase in the ratio between the cortex and medulla, favoring the development and proliferation of B lymphocytes within the lymphoid follicles. Positive effects of the G-GxB-BL lights were also observed through the maintenance of a columnar morphological shape of the epithelium associated with lymphoid follicles (FAE), suggesting optimal specialization for antigen capture and processing, which is essential in the immune education of B lymphocytes. The activity of B lymphocytes in BF was mediated by G-GxB-BL lights, which caused a significant increase in the number of cells that were positive for Mel1a and PCNA and a significant decrease in cells that were positive for RORα. There were early signs of the physiological involution of BF, with a decrease in the ratio between the cortex and medulla, the blurring of the clear boundary between the two areas, a tendency toward flattening of the FAE epithelium, a decrease in the number of Mel1a- and PCNA-positive cells, and an increase in RORα-positive cells. The G-GxB-BL lights delayed the onset of these signs and practically supported more active immune activity of the BF. This research was conducted based on the multitude of studies related to the modulation of the immune system in animals by colored lights. Our research adds new information to the results that are already available in the literature, and the immunohistochemical analysis provides information on the quality and quantity of the immune marker in the BF, while the histological analysis refers to complex morphometric indices that reflect in depth the structural changes that express the immune activity of BF. The present study is limited, and we propose that future studies address aspects related to the behavior of chicks under different types and colors of light, the dosage of serum melatonin, and the analysis of its anti-inflammatory and antioxidant power, as well as the intracellular signaling pathways of melatonin receptors. This study can provide appropriate guidance for its application in the modern broiler industry to improve the growth performance and immune status in broilers.

## 5. Conclusions

Overall, our results show that exposure to green light in the first period of life, then a combination of green and blue light in the first growth period, and only blue light in the last growth period of broiler chickens, improves ADFI and FCR, the bursa index and the lymphoid follicle area. This combination of lights also improved B-lymphocyte proliferation in the BF by significantly increasing the lymphocyte density and the cortico-medullary ratio, as well as the number of PCNA-positive cells. The activation and modulation of the immune system by these lights was achieved by activating melatonin receptors, leading to a significant increase in the number of Mel1a-positive cells and a decrease in RORα-positive cells. However, the mechanism by which colored lights influence melatonin synthesis and the power of this hormone in phototransduction and immune regulation remains a subject for future research.

## Figures and Tables

**Table 1:** Light parameters.

Light Treatments	0 Days–14 Days	15 Days–28 Days	35 Days–42 Days
Light wavelength (nm) ^1^	400–760	400–760	400–760
Light wavelength (nm) ^2^	560	480–560	480
Light intensity (W/m^2^)	0.19	0.19	0.19
Photoperiod (light:dark)	23:1	16:8	16:8

^1^ WL group and ^2^ G-GXB-BL group.

**Table 2:** The composition of starter, grower and finisher diets for broilers (%).

Ingredient (%)	Starter (1–10 Days)	Grower (11–24 Days)	Finisher (25–42 Days)
Corn	52.50	55.00	61.00
Soybean meal	38.00	31.50	25.00
Wheat	3.00	7.00	7.00
Soy oil	3.00	3.00	3.00
Limestone	1.35	1.15	1.10
Dicalcium phosphate	1.25	1.00	0.80
L-Lysine	0.30	0.20	0.10
DL-Methionine	0.20	0.10	0.05
L-Threonine	0.10	0.05	0.03
Vitamin mix ^1^	0.10	0.35	0.81
Mineral mix ^2^	0.10	0.35	0.81
Salt	0.10	0.30	0.30
Chemical composition			
ME (kcal/kg)	3015.00	3150.00	3250.00
Protein (%)	23.25	21.00	19.50
Lysine (%)	1.35	1.15	1.05
Methionine + Cystein (%)	1.00	0.85	0.80
Calcium (%)	1.05	0.95	0.90
Available phosphorus (%)	0.50	0.45	0.40

^1^ Vitamin mix provided per kilogram of diet: vitamin A: 9000 IU; vitamin D3: 2400 IU; vitamin E: 18 IU; choline chloride: 500 mg; vitamin K: 2 mg; vitamin B1: 18 mg; vitamin B2: 6.6 mg; vitamin B3: 10 mg; vitamin B5: 4.8 mg; vitamin B6: 3 mg; vitamin B7: −0.15 mg; vitamin B9: 1 mg; vitamin B12: 0.015 mg and ethoxyquin: 1 mg. ^2^ Mineral mix provided per kilogram of diet: Fe: 50 mg; Cu: 10 mg; Zn: 7.84 mg; Se: 0.2 mg; Mn: 100 mg and Iodine: 1 mg.

**Table 3:** The antibodies used in the experiment.

Name	Cat. No.	Dilution	Host	Manufacturer
Primary antibodies				
PCNA	PA5-2714	1:100	rabbit	Invitrogen
Mel1a	orb221456	1:100	rabbit	Biorbyt
RORα	AA 239-288	1:50	rabbit	Antibodies online
Secondary antibodies				
Goat Anti-Rabbit	No RE7140K Kit	1:100	rabbit	Leica Biosystems

**Table 4:** The effects of the combination of green and blue monochromatic lights on the growth performance of male and female broilers within the age range of 7–42 days.

Period	WL	G-GxB-BL	SEM	P-Light	P-Sex	P-L × S	η^2^
Body weight (g)							
7 Days	160.66	163.94	5.751	0.579	<0.001	0.873	0.026
14 Days	470.31	478.70	12.574	0.517	0.009	0.539	0.036
21 Days	971.00	1020.67	25.372	0.074	0.009	0.949	0.242
28 Days	1455.71	1551.08	28.734	0.006	<0.001	0.718	0.479
35 Days	2235.54	2326.09	81.626	0.289	<0.001	0.615	0.093
42 Days	2861.67	2996.09	73.063	0.091	<0.001	0.157	0.220
ADG (g/bird/day)							
7 Days	22.95	24.14	1.117	0.307	<0.001	0.602	0.087
14 Days	33.59	34.19	0.897	0.517	0.009	0.538	0.036
21 Days	46.24	48.60	1.209	0.074	0.009	0.951	0.242
28 Days	51.99	55.39	1.026	0.006	<0.001	0.717	0.479
35 Days	63.97	66.46	2.332	0.289	<0.001	0.616	0.093
42 Days	68.13	71.33	1.738	0.091	<0.001	0.158	0.220
ADFI (g/bird/day)							
7 Days	26.16	25.48	0.281	0.032	0.001	0.658	0.328
14 Days	53.30	52.81	0.276	0.102	<0.001	0.996	0.207
21 Days	90.68	89.71	0.285	0.005	<0.001	0.576	0.490
28 Days	131.23	130.77	0.339	0.195	<0.001	0.840	0.136
35 Days	169.73	169.22	0.326	0.142	<0.001	0.967	0.170
42 Days	201.03	200.35	0.211	0.008	<0.001	0.474	0.461
FCR (g/g)							
7 Days	1.17	1.13	0.039	0.263	<0.001	0.926	0.103
14 Days	1.60	156	0.041	0.375	0.136	0.475	0.066
21 Days	1.98	1.85	0.052	0.034	0.440	0.981	0.323
28 Days	2.56	2.39	0.054	0.008	0.050	0.421	0.459
35 Days	2.78	2.60	0.168	0.318	0.061	0.942	0.083
42 Days	3.01	2.82	0.090	0.059	0.451	0.126	0.267
Mortality (%)							
7 Days	0.74	0.30	0.228	0.175	0.640	0.640	0.148
14 Days	0.30	0.00	0.201	0.183	0.995	0.995	0.143
21 Days	0.00	0.00	0.00	N.a.	N.a.	N.a.	N.a.
28 Days	0.00	0.00	0.00	N.a.	N.a.	N.a.	N.a.
35 Days	0.00	0.00	0.00	N.a.	N.a.	N.a.	N.a.
42 Days	0.00	0.00	0.00	N.a.	N.a.	N.a.	N.a.
Flock uniformity (CV %)							
7 Days	9.08	5.50	2.343	0.152	0.470	0.743	0.136
14 Days	4.58	8.90	1.922	0.044	0.825	0.735	0.296
21 Days	8.03	5.75	2.160	0.312	0.424	0.288	0.085
28 Days	11.77	9.03	3.219	0.411	0.271	0.633	0.057
35 Days	8.38	7.67	3.871	0.857	0.558	0.272	0.003
42 Days	8.96	5.31	3.314	0.292	0.600	0.452	0.092

The main effects and interactions were calculated using two-way ANOVA; the overall consistency of the growth patterns was verified through repeated measures ANOVA. The experimental unit was n = 4 pens/light treatment/sex. Abbreviations: SEM—standard error of mean (n = 16 pens), P-L × S—interaction between lighting treatment and sex, η^2^—partial eta squared (effect size for the main effect of light), ADG—average daily gain; ADFI—average daily feed intake, FCR—feed conversion ratio and N.a.—not applicable.

## Data Availability

All data generated or analyzed during this study are included in this published article and its Supplementary Materials.

## Supplementary Materials

The following supporting information can be downloaded at: https://www.mdpi.com/article/10.3390/ani16081238/s1, Table S1: The effects of the combination of green and blue monochromatic lights on BF development of male and female broilers within the age range of 7–42 days; Table S2: The effects of the combination of green and blue monochromatic lights on PCNA, Mel1a and RORα Expression in BF of male and female broilers within the age range of 7–42 days.
