# Supplementary material for: The Effects of a Mixture of Monochromatic Green and Blue Light on Growth Performance and Immune Response in Bursa of Fabricius by Morphometry Using Staining and Immunohistochemistry in Broiler Chickens
Source: Animals (Basel). 2026 Apr 17;16(8):1238. doi: 10.3390/ani16081238 (PMC13114016; doi:10.3390/ani16081238)
Supplement: Supplementary file 1 [file animals-16-01238-s001.zip › animals-4216883-supplementary.pdf]

# Supplementary materials

## 1. Supplementary tables

**Supplementary Table S1.** The effects of the combination of green and blue monochromatic lights on BF development of male and female broilers within the age range of 7-42 days.

| Period | WL | G-GxB-BL | SEM | P-Light | P-Sex | P- L*S | $\eta^2$ |
|---|---|---|---|---|---|---|---|
| Index organ | | | | | | | |
| 7 Days | 0.20 | 0.26 | 0.002 | <0.001 | <0.001 | 0.003 | 0.983 |
| 14 Days | 0.22 | 0.24 | 0.001 | <0.001 | <0.001 | <0.001 | 0.985 |
| 21 Days | 0.20 | 0.21 | 0.002 | <0.001 | 0.022 | 0.052 | 0.734 |
| 28 Days | 0.14 | 0.17 | 0,002 | <0.001 | <0.001 | 0.026 | 0.967 |
| 35 Days | 0.06 | 0.07 | 0.001 | <0.001 | 0.013 | 0.104 | 0.804 |
| 42 Days | 0.44 | 0.05 | 0.001 | <0.001 | 0.001 | 0.739 | 0.699 |
| Area of lymphoid follicles | | | | | | | |
| 7 Days | 0.03 | 0.05 | 0.001 | <0.001 | <0.001 | 0.223 | 0.975 |
| 14 Days | 0.03 | 0.06 | 0.001 | <0.001 | <0.001 | <0.001 | 0.979 |
| 21 Days | 0.07 | 0.09 | 0.001 | <0.001 | <0.001 | 0.001 | 0.974 |
| 28 Days | 0.07 | 0.09 | 0.003 | <0.001 | 0.004 | 0.884 | 0.816 |
| 35 Days | 0.09 | 0.12 | 0.001 | <0.001 | <0.001 | 0.299 | 0.993 |
| 42 Days | 0.10 | 0.13 | 0.001 | <0.001 | <0.001 | <0.001 | 0.989 |
| Cortex/ medulla | | | | | | | |
| 7 Days | 0.83 | 0.91 | 0.010 | <0.001 | <0.001 | 0.089 | 0.834 |
| 14 Days | 1.06 | 1.25 | 0.011 | <0.001 | 0.016 | 0.369 | 0.965 |
| 21 Days | 1.47 | 1.59 | 0.012 | <0.001 | <0.001 | <0.001 | 0.902 |
| 28 Days | 1.14 | 1.31 | 0.012 | <0.001 | <0.001 | <0.001 | 0.942 |
| 35 Days | 0.95 | 1.01 | 0.009 | <0.001 | <0.001 | 0.013 | 0.804 |
| 42 Days | 0.66 | 0.75 | 0.011 | <0.001 | <0.001 | <0.001 | 0.855 |
| Density | | | | | | | |
| 7 Days | 1.44 | 1.47 | 0.006 | <0.001 | <0.001 | <0.001 | 0.664 |
| 14 Days | 1.53 | 1.61 | 0.007 | <0.001 | <0.001 | 0.003 | 0.913 |
| 21 Days | 1.62 | 1.64 | 0.005 | 0.006 | <0.001 | 0.036 | 0.476 |
| 28 Days | 1.66 | 1.68 | 0.008 | 0.004 | 0.350 | 0.007 | 0.515 |
| 35 Days | 1.70 | 1.73 | 0.007 | 0.001 | 0.086 | 0.161 | 0.626 |
| 42 Days | 1.79 | 1.77 | 0.007 | 0.050 | <0.001 | 0.419 | 0.283 |
| Height of FAE | | | | | | | |
| 7 Days | 22.74 | 23.91 | 1.925 | 0.553 | 0.156 | 0.704 | 0.030 |
| 14 Days | 27.89 | 30.08 | 1.709 | 0.223 | 0.067 | 0.857 | 0.121 |
| 21 Days | 35.67 | 42.58 | 1.206 | <0.001 | 0.005 | 0.001 | 0.732 |
| 28 Days | 36.02 | 48.21 | 1.381 | 0.000 | 0.007 | 0.108 | 0.867 |
| 35 Days | 28.51 | 32.90 | 3.672 | 0.256 | 0.073 | 0.510 | 0.106 |
| 42 Days | 25.49 | 26.48 | 1.010 | 0.346 | 0.014 | 0.117 | 0.074 |

**Supplementary Table S2.** The effects of the combination of green and blue monochromatic lights on PCNA, Mel1a and ROR$\alpha$ Expression in BF of male and female broilers within the age range of 7-42 days.

| | WL | G-GxB-BL | SEM | P-Light | P-Sex | P- L*S | $\eta^2$ |
|---|---|---|---|---|---|---|---|
| PCNA | | | | | | | |
| 7 Days | 8.55 | 10.12 | 0.041 | <0.001 | <0.001 | <0.001 | 0.992 |
| 14 Days | 9.39 | 10.15 | 0.066 | <0.001 | <0.001 | <0.001 | 0.915 |
| 21 Days | 10.48 | 12.63 | 0.169 | <0.001 | <0.001 | <0.001 | 0.931 |
| 28 Days | 10.54 | 13.40 | 0.115 | <0.001 | <0.001 | <0.001 | 0.981 |
| 35 Days | 5.67 | 8.17 | 0.040 | <0.001 | <0.001 | <0.001 | 0.997 |
| 42 Days | 5.32 | 7.94 | 0.103 | <0.001 | <0.001 | <0.001 | 0.982 |
| Mel1a | | | | | | | |
| 7 Days | 5.49 | 9.69 | 0.122 | <0.001 | <0.001 | <0.001 | 0.990 |
| 14 Days | 6.33 | 11.72 | 0.164 | <0.001 | <0.001 | <0.001 | 0.989 |
| 21 Days | 9.47 | 12.42 | 0.131 | <0.001 | <0.001 | 0.001 | 0.977 |
| 28 Days | 9.88 | 13.06 | 0.163 | <0.001 | <0.001 | 0.013 | 0.969 |
| 35 Days | 2.46 | 5.43 | 0.082 | <0.001 | <0.001 | <0.001 | 0.991 |
| 42 Days | 0.16 | 0.58 | 0.020 | <0.001 | <0.001 | <0.001 | 0.974 |
| RORa | | | | | | | |
| 7 Days | 0.56 | 0.12 | 0.017 | <0.001 | <0.001 | 0.003 | 0.982 |
| 14 Days | 0.68 | 0.22 | 0.016 | <0.001 | <0.001 | 0.288 | 0.986 |
| 21 Days | 1.42 | 0.97 | 0.013 | <0.001 | <0.001 | <0.001 | 0.990 |
| 28 Days | 1.57 | 1.14 | 0.011 | <0.001 | <0.001 | 0.746 | 0.992 |
| 35 Days | 2.58 | 1.82 | 0.043 | <0.001 | <0.001 | <0.001 | 0.964 |
| 42 Days | 2.98 | 1.96 | 0.030 | <0.001 | <0.001 | <0.001 | 0.990 |
